# Essential Oils as Antimicrobials in Crop Protection

**DOI:** 10.3390/antibiotics10010034

**Published:** 2021-01-01

**Authors:** María Alonso-Gato, Gonzalo Astray, Juan C. Mejuto, Jesus Simal-Gandara

**Affiliations:** 1 Department of Physical Chemistry, Faculty of Science, University of Vigo–Ourense Campus, E32004 Ourense, Spain; mariaalonsogato@gmail.com (M.A.-G.); xmejuto@uvigo.es (J.C.M.); 2 CITACA, Agri-Food Research and Transfer Cluster, University of Vigo, 32004 Ourense, Spain; 3 Nutrition and Bromatology Group, Department of Analytical and Food Chemistry, Faculty of Food Science and Technology, University of Vigo–Ourense Campus, E32004 Ourense, Spain

**Keywords:** essential oil, antibacterial, antifungal, crop protection

## Abstract

At present, organic crops have reached an important boom in a society increasingly interested in the conservation of the environment and sustainability. It is evident that a part of the population in the Western world focuses their concern on how to obtain our food and on doing it in a way that is as respectful as possible with the environment. In this review, we present a compilation of the work carried out with the use of essential oils as an alternative in the fight against different bacteria and fungi that attack crops and related products. Given the collected works, the efficacy of essential oils for their use as pesticides for agricultural use is evident.

## 1. Introduction to Essential Oils

Essential oil is a term reserved for those compounds that are defined by the International Organization for Standardization (ISO) [1] in their ISO 9235 [2]. These kinds of compounds are complex mixtures originated from the secondary metabolism [3,4], produced by the glandular trichomes, and in different secretory structures [4,5]. They can be composed by terpenes, associated or not to other components, generally volatile and that provides an odor to the vegetable [6]. These compounds have (with exceptions) a density lower than water density [5,6] and are usually presented in liquid form [5,6]. Besides, essential oils are hydrophobic compounds, soluble in alcohol (among others) and only a little soluble in water [5].

According to the ISO 9235 from the International Organization for Standardization (ISO), the essential oil can be obtained by distillation by any of its variants: hydrodistillation, steam distillation, or dry distillation and by mechanical processes [2]. Some of these distillation methods are widely reported in the literature [7,8,9,10], even some variants such as distillation with cohobation [8] have been reported. Because essential oils are responsible for the aroma of plants, they are widely known for their use in cosmetics and perfumery, but they are also an important resource in other industrial fields such as pharmaceutical, food, among others [1,4,5,11]. Indeed, and according to Turek and Stintzing [1], they are a viable environmental-friendly alternative in these fields due to their proved capacity as nematicidal [12,13], antimicrobial [14,15], insecticidal [16], antifungal [15,17] or, even, herbicidal and insect repellent [18]. Besides this, essential oils show antioxidant activity (that can be used in edible products or active packaging) [19], anticancer properties [20], and properties for pain or inflammation treatment [21].

**Figure 1:**
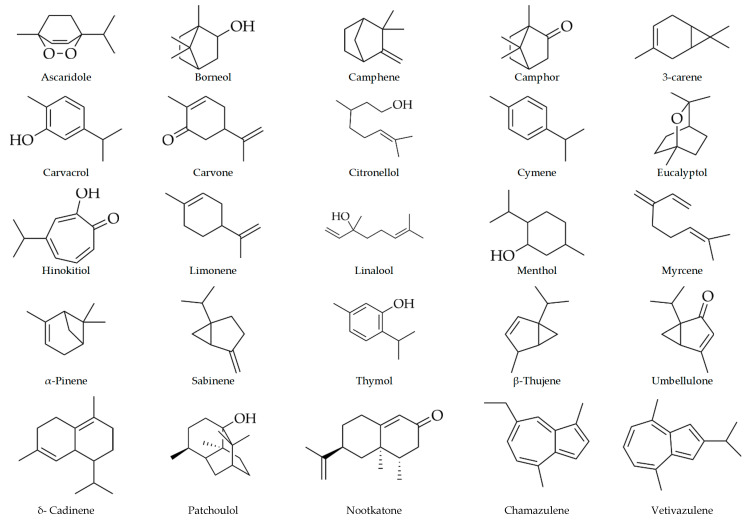
Some monoterpenoids and sesquiterpenoids presented in essential oils.

Chemically, essential oils are complex mixtures of more than 100 components [5,11,22], but they are mainly made up of terpenic compounds [23]. Terpenoids, sometimes called isoprenoids, are a broad family of natural compounds derived from isoprene [24]. About 60% of known natural products are terpenoids [25]. This class of secondary compounds (essential oils) contains different terpenoids such as monoterpenes (C_10_) [18,26] contain two isoprene units (linear or cyclic [26]) like myrcene, menthol, limonene, or linalool (see Figure 1). Other important components of essential oils are the sesquiterpenes which consist of three isoprene units (C_15_) [18,26]. Figure 1 shows some different sesquiterpenes such as patchoulol or nootkatone (see Figure 1). According to the information reported by Martinez (2003) [11], monoterpenoids are common in the Primulales, Ranunculales, and Violales orders (being scarcer in other different orders such as Asterales, Cornales, Lamiales, and Rutales) and sesquiterpenoids are mainly abundant in other orders such as Asterales, Cornales, Magnoliales, and Rutales [11]. Essential oils can also contain diterpenes (as by-product) [26] which are composed of four isoprene units -as retinal or phytol-. Finally, essential oils contain other compounds such as aromatic phenols, ethers, esters, alcohols, among others, which will confer the aroma and odor of the plant [18].

Essential oil components can convert without difficulty into each other by a different process (cyclization, isomerization, oxidation, among other ways) due to their structural relation inside the same chemical group [1].

The chemical composition of essential oils depends on different factors such as plant’s physiology, climate characteristics or, even, soil conditions where the plant grows [3]. According to this, within the same plant species, or even in their different organs, the chemical composition may vary [3]. The chemical composition can also be influenced by plant health or harvest time [1].

According to Montoya Cadavid [27], around 60 and 80 families produce essential oils, the largest number grows in tropical climates, although they are also found in other climates, with the spermatophytes as the main plants that produce essential oils. Essential oils are found in the different organs of the plant [11,27]: roots (e.g., turmeric, saffron, ginger, and sandalwood), flowers (e.g., thyme, lavender, arnica, and chamomile), fruits (e.g., laurel, coriander, parsley, or pepper), and in other parts such as seeds or leaves, among others.

## 2. Essential Oils as Antibacterial and Antifungal Compounds

Many secondary metabolites produced by plants can present an important role in their protection against microbes potentially pathogenic [4]. These compounds are a very attractive alternative to many antibiotics due to the bacterial resistance generated to traditional products [4]. An example of this is the use of genus Origanum or Thymus due to their antimicrobial activity. However, many times these studies are only focused on the antibacterial activities and do not enter to assess the action mechanism [4].

The following sections present plants whose essential oils have toxic action against a certain organism. The classification contains bacteria and fungi that can affect crop production and other plants and products of interest.

### 2.1. Antibacterial Activity

Many pathogenic bacteria in crops can cause serious problems and symptoms in the different parts of the plants such as spots, cankers, rots, wilting, among others [28,29,30,31,32]. Different studies have shown that essential oils present antibacterial properties against pathogens which can cause post-harvest diseases in vegetables and fruits [32]. In fact, the use of plants, herbs, or spices (for medical, preservatives, and/or pest control) has been reported since ancient civilizations of Egypt, Greece, Rome, among others [33,34,35] and their properties have been systematically analyzed in laboratories since the beginning of the last century [36,37], and even before. Besides, they have demonstrated their efficacy against multidrug-resistant/antibiotic-resistant bacteria [38,39].

Essential oils present different components and it is possible that their action implicates different targets in the bacteria cell [40]. Their cytotoxic properties are due to the disruption of membranes structure that results in bacterial cell permeabilization so different cellular functions (membrane potential, among others) are altered [4]. It can be said that their antimicrobial activity is due to the solubility of essential oils in the bilayer [41].

The use of essential oils (or their respective plants of origin) to combat this type of organism is not a minor issue, due to bacteria leading different diseases on plants which can have a notable economic impact [42]. Due to this, in recent years, numerous studies have been carried out on the antibacterial properties of essential oils against different pathogens that can attack plants, and even food [42].

Table 1 shows a compilation of research articles [43,44,45,46,47,48,49,50,51,52,53,54,55,56,57] in which different plants/essential oils were analyzed by their possible antibacterial activity that can affect both crops and stored products, among others. This table contains part of the experimental work developed by the researchers (for a better understanding, consult the original sources). All these studies can be the initial step for the development of new products to combat these types of pathogens.

### 2.2. Antifungal Activity

Essential oils have also been presented in antifungal properties. The fungal diseases can have different consequences on plants such as alterations in their physiology, disturb their usual functioning, reduce their performance and, even, sometimes the loss of the plant [58]. Different essential oils from plants (and their dominant constituents) have proved antifungal activity against different plant pathogenic fungi [59]. According to Isman and Machial [59], their action mechanisms is unknown but can be linked to their capacity to dissolve/disrupt the integrity of the fungi’s membranes and cell walls. According to Arraiza et al. [58], in different studies, the antifungal properties are based on the inhibition of fungal mycelial growth in vitro. However, it is necessary to be careful with the use of essential oils because many of these oils (and their pure compounds) can present high phytotoxicity for plants (even in concentrations slightly higher than those necessary for the control of fungi), possibly due to plant cells being affected by the same mechanism [59].

Table 2 shows a compilation of research articles [53,60,61,62,63,64,65,66,67,68,69,70,71,72] in which different plants/essential oils were analyzed by their possible antifungal activity. As previously said, this table contains only a part of the experimental work (for a better understanding, consult the original sources).

## 3. Advantages and Drawbacks of Essential Oils Based Biopesticide for Crop Protection Control

Many studies have shown that different constituents of essential oils can present antibacterial and antifungal properties so that there are real possibilities to used essential oils for plants and crop protection. These kinds of products, made based on essential oils, could be considered as biopesticides. However, different authors suggest that the term “biopesticide” should be reserved only for living organisms (biological agents) [73,74]. This definition is too restrictive and would not include different products derived from the metabolism of the biological organisms [73,74] (plants in our case). Thus, in the current crop protection situation, a wide definition of biopesticides could encompass all compounds of biological origin and seem more suitable [73].

Essential oils-based biopesticides present some advantages for crop protection. Numerous essential oils, that have a large number of plants, cover a wide spectrum of activities against pest insects and pathogenic fungi [75]. These compounds show highly useful against a broad range of agricultural pests and diseases [76]. They present a low persistence in the medium due to its high volatility [75] so that they produce little or no toxic residue and as a result, they do not pollute the soil or groundwater. Besides this, the most essential oils are reasonably nontoxic to mammals and aquatic life and they can be classified as low-risk pesticides [75], in other words, essential oils could present low toxicity against non-target organisms [76]. Another advantage of this group of compounds is that some essentials oils are available in large measure, due to this, the commercialization of pesticides based on essential oils is possible [75], besides, for its application, the same spray equipment can be often used [77] which means that the end-user should not acquire any new equipment for the use of these biopesticides.

On the other hand, and due to that essential oils are a complex mixture of compounds, the possible development of resistance by the pest is slow [75] or less probable [76]. Finally, the use of biopesticides frequently presents good compatibility with conventional chemical pesticides and with biological pest control agents [77].

Although essential oils (and biopesticides in general) have shown many advantages over the use of traditional/conventional pesticides, they present several disadvantages. At the commercial level, not many kinds of this product have arisen in the market, probably due to the high cost of necessary evaluations (toxicology and environmental) [75] because the authorization processes of these biopesticides (botanical pesticides) are complex [76]. Furthermore, according to Isman [78], it would be necessary to have sufficient availability, uniformity, and purification technology protection and a homologation following a regulatory framework. Besides, essential oils are a complex mixture of compounds (beneficial effect to slow down resistance [75,76]), nevertheless, the characterization and specificity detection of each compound that constitutes the essential oil is inaccessible for their use in agricultural farms [75].

On the other hand, some biopesticides can show low persistence (something positive) but they can also be considered as a drawback after application [79] because essential oils can suffer gradual biodegradation of their active substances after application [76]. Related to this, essential oil-based pesticides present less effective when they are compared with other synthetic/conventional chemical pesticides [75,77]. Finally, these kinds of pesticides generally require higher application rates, which together with the need to apply the product frequently, makes their use expensive and time-consuming [75].

To finish, Pavela and Benelli [76] reported that there are numerous studies centered on the biological activity of essential oils on target organisms, nevertheless, it would be necessary to further research toxicological studies and the possible effects of their use on non-target ones. Furthermore, according to the same authors [76], the mechanisms of action and other properties of interest have not yet been clarified. Despite this, the authors report that based on the existing toxicological studies, it can be concluded that the most essential oils can be considered safe (for human and the environment) in the concentrations or doses commonly used, and based on this, the legislation could be simplified and establish a greater partnership between research and the manufacturers of botanical pesticides [76].

## 4. Conclusions

It seems clear that there are many and different characteristics that can be attributed to essential oils. All of these comes from studies developed in recent decades that have been carried out based on their biological activity and their use in different fields such as agronomy and food production. These studies have shown the relative efficacy of the use of the essential oils against different types of organisms, their action mechanisms, and their low toxicity in mammals and humans.

Their possible use presents, as expected, advantages for the environment such as their volatility (less persistent than other synthetic chemicals) or that they are a new alternative to the resistance to synthetic chemicals by organisms. As main drawbacks, those related to their availability, the high cost of the authorization processes, their low persistence, and less effective action. On the other hand, it is necessary to determine their optimal ratio and dosages to improve their efficacy and decrease their toxicity, all these aimed to have sufficient scientific information to be able to be safely marketed.

Essential oils could mean the appearance of a new era of plant protection products to control the microbial pathogens and prevent their propagation and resistance.

## Figures and Tables

**Table 1:** Plants that present essential oils that could be used for their antibacterial properties.

Plant			
Common Name	Scientific Name	Organism to Fight against	Ref.
Sandalwood	*Amyris balsamifera*	*Xylella fastidiosa*	[43]
Dill	*Anethum graveolens*	*Streptomycetes scabies*	[44]
Caraway	*Carum carvi* L.	*Agrobacterium tumefaciens*	[45]
		*Bacillus megaterium*	
		*Clavibacter michiganensis* subsp. *michiganensis*	
		*Clavibacter michiganensis* subsp. *sepedonicus*	
		*Curtobacterium flaccumfaciens* pv. *betae*	
		*Curtobacterium flaccumfaciens* pv. *flaccumfaciens*	
		*Erwinia carotovora* subsp. *atroseptica*	
		*Ralstonia solanacearum*	
		*Xanthomonas campestris* pv. *campestris*	
		*Xanthomonas campestris* pv. *phaseoli*	
		*Xanthomonas campestris* pv. *phaseoli var. fuscans*	
		*Xanthomonas campestris* pv. *vesicatoria*	
*Cinnamon*	*Cinnamomum verum*	*Acidovorax citrulli*	[46]
		*Ralstonia solanacearum*	[47]
Cumin	*Cuminum cyminum* L.	*Agrobacterium tumefaciens*	[45]
		*Clavibacter michiganensis subsp. michiganensis*	
		*Clavibacter michiganensis* subsp. *sepedonicus*	
		*Curtobacterium flaccumfaciens* pv. *betae*	
		*Curtobacterium flaccumfaciens* pv. *flaccumfaciens*	
		*Erwinia carotovora* subsp. *atroseptica*	
		*Ralstonia solanacearum*	
		*Rhodococcus fascians*	
		*Xanthomonas campestris* pv. *phaseoli*	
		*Xanthomonas campestris* pv. *phaseoli* var. *fuscans*	
		*Xanthomonas campestris* pv. *vesicatoria*	
Palmarosa	*Cymbopogon martini*	*Ralstonia solanacearum* Race 4	[48]
Lemongrass	*Cymbopongo citratus*		
Common juniper	*Juniperus communis*	*Enterococcus faecalis*	[49]
		*Listeria monocytogenes*	
		*Staphylococcus aureus*	
Cade juniper	*Juniperus oxycedrus*	*Enterococcus faecalis*	
		*Listeria monocytogenes*	
		*Staphylococcus aureus*	
Common lantana	*Lantana camara*	*Ralstonia solanacearum* phylotype II	[50]
Lemon blam	*Melissa officinalis*	*Pantoea agglomerans*	[51]
		*Pseudomonas fluorescens*	
		*Pseudomonas syringae* pv. *syringae*	
Corn mint	*Mentha arvensis*	*Erwinia amylovora*	
		*Pantoea agglomerans*	
		*Pseudomonas syringae* pv. *syringae*	
*Peppermint*	*Mentha piperita*	*Acidovorax citrulli*	[46]
Mint	*Mentha spicata*	*Bacillus subtilis*	[52]
		*Erwinia carotovora*	
		*Escherichia coli*	
		*Klebsiella pneumoniae*	
		*Staphylococcus aureus*	
		*Xanthomonas campestris*	
Catnip	*Nepeta cataria*	*Erwinia amylovora*	[51]
		*Pantoea agglomerans*	
*Sweet basil*	*Ocimum basilicum*	*Acidovorax citrulli*	[46]
	*Origanum compactum*	*Erwinia amylovora*	[51]
		*Pantoea agglomerans*	
		*Pantoea dispersa*	
		*Pseudomonas fluorescens*	
		*Pseudomonas syringae* pv. *syringae*	
Dictamnus	*Origanum dictamnus*	*Clavibacter michiganensis* subsp. *michiganensis*	[53]
Marjoram	*Origanum majorana*	*Clavibacter michiganensis* subsp. *michiganensis*	
Origanum	*Origanum majorana* L.	*Acidovorax avenae* subsp. *citrulli*	[54]
Oregano	*Origanum onites*	*Streptomycetes scabies*	[44]
Oregano	*Origanum vulgare*	*Clavibacter michiganensis* subsp. *michiganensis*	[53]
		*Erwinia amylovora*	[51]
		*Pantoea agglomerans*	
		*Pantoea dispersa*	
		*Pseudomonas fluorescens*	
		*Pseudomonas syringae* pv. *syringae*	
Patchouli	*Pogostemon patchouli*	*Xylella fastidiosa*	[43]
	*Satureja adamovicii* Šilić	*Erwinia amylovora*	[55]
	*Satureja fukarekii* Šilić		
Summer savory	*Satureja hortensis*		[56]
	*Satureja kitaibelii* Wierzb. ex Heuff.		[55]
	*Satureja montana* ssp. *montana* L.		
*Clove bud*	*Syzygium aromaticum*	*Acidovorax citrulli*	[46]
Thyme	*Thymbra spicata* L. subsp. *spicata*	*Acidovorax avenae* subsp. *citrulli*	[54]
Thyme	*Thymus capitatus*	*Clavibacter michiganensis* subsp. *michiganensis*	[53]
Thyme	*Thymus serpyllum* L.	*Acidovorax avenae* subsp. *citrulli*	[54]
Thyme	*Thymus vulgaris*	*Erwinia amylovora*	[51]
			[56]
		*Pantoea agglomerans*	[51]
		*Pantoea dispersa*	
		*Pseudomonas fluorescens*	
		*Pseudomonas syringae* pv. *syringae*	
Ajowan	*Trachyspermum ammi*	*Erwinia carotovora*	[57]

**Table 2:** Plants and essential oils that could be used for their antifungal properties.

Plant			
Common Name	Scientific Name	Organism to Fight against	Ref.
	*Cinnamomum camphora* var. *Linaloolifera*	*Alternaria solani*	[60]
Cinnamon bark oil	*Cinnamomum cassia*	*Villosiclava virens*	[61]
Cinnamon oil			
Gingergrass	*Cymbopogon martinii*	*Fusarium graminearum*	[62]
	*Cymbopogon martinii* (chitosan nanoparticles)		
Lemongrass	*Cympopogon citratus* L.	*Botrytis cinerea*	[63]
		*Cladosporium herbarum*	
		*Colletotrichum coccodes*	
		*Rhizopus stolonifer*	
River red gum	*Eucalyptus camaldulensis* Dehnh.	*Fusarium oxysporum*	[64]
		*Fusarium proliferatum*	
		*Fusarium solani*	
		*Fusarium subglutinans*	
		*Fusarium verticillioides*	
Eucalyptus	*Eucalyptus citriodora*	*Venturia inaequalis*	[65]
Southern blue gum	*Eucalyptus globulus*	*Alternaria solani*	[60]
Lemon-scented ironbark	*Eucalyptus staigeriana*		
Espliego	*Lavandula latifolia* Medik.	*Fusarium moniliforme*	[66]
		*Fusarium oxysporum*	
		*Fusarium solani*	
Lavandín	*Lavandula* x intermedia Emeric ex Loisel.	*Fusarium moniliforme*	
		*Fusarium oxysporum*	
		*Fusarium solani*	
Prontoalivio	*Lippia alba* (Mill.) N.E. Brown (Chemotype Caxias do Sul)	*Alternaria solani* (Pleosporeaceae)	[67]
	*Lippia alba* (Mill.) N.E. Brown (Chemotype Pelotas)		
	*Lippia alba* (Mill.) N.E. Brown (Chemotype Santa Vitória do Palmar)		
	*Lippia alba* (Mill.) N.E. Brown (Chemotype Teutônia)		
Tea tree	*Melaleuca alternifolia*	*Blumeria graminis*	[68]
		*Fusarium culmorum*	
		*Fusarium graminearum*	
		*Pyrenophora graminea*	
White basil	*Ocimum basilicum L.*	*Alternaria solani Sorauer*	[69]
Genovese basil	*Ocimum basilicum L.* var. *Genovese*		
	*Ocotea quixos* (Lam.) Kosterm	*Aspergillus oryzae*	[70]
		*Cladosporium cladosporioides*	
		*Fusarium solani*	
		*Moniliophthora roreri*	
		*Phytophthora* sp.	
		*Rhizopus stolonifer*	
Dictamnus	*Origanum dictamnus*	*Botrytis cinerea*	[53]
		*Fusarium solani var. coeruleum*	
Marjoram	*Origanum majorana*	*Botrytis cinerea*	
		*Fusarium solani var. coeruleum*	
Oregano	*Origanum vulgare*	*Botrytis cinerea*	
		*Fusarium solani var. coeruleum*	
Jamaica pepper	*Pimenta dioica* (L.) Merr.	*Aspergillus flavus*	[71]
		*Aspergillus fumigatus*	
		*Fusarium oxysporum*	
		*Fusarium verticillioides*	
		*Penicillium brevicompactum*	
		*Penicillium expansum*	
Anise	*Pimpinella anisum* L.	*Alternaria solani Sorauer*	[69]
Spiked pepper	*Piper aduncum* L.	*Fusarium solani*	[70]
		*Phytophthora* sp.	
Caisimón de anís	*Piper auritum* Kunth	*Alternaria solani Sorauer*	[69]
Clove	*Syzygium aromaticum*	*Penicillium digitatum Sacc.*	[72]
		*Venturia inaequalis*	[65]
Thyme	*Thymus capitatus*	*Botrytis cinerea*	[53]
		*Fusarium solani var. coeruleum*	
	*Thymus vulgaris* L.	*Fusarium moniliforme*	[66]
		*Fusarium oxysporum*	
		*Fusarium solani*	

## Data Availability

All data were properly cited.

## References

[1] Turek C., Stintzing F.C. (2013). Stability of Essential Oils: A Review. Comp. Rev. Food Sci. Food Saf..

[2] ISO 9235 (2013). Aromatic Natural Raw Materials—Vocabulary.

[3] Espitia-Yanes C.R. (2011). Evaluación de la Actividad Repelente e Insecticida de Aceites Esenciales Extraídos de Plantas Aromáticas (Cymbopogon Citratus y Tagetes Lucida) Utilizados Contra Tribolium Castaneum Herbst. (Coleoptera: Tenebrionidae). Master’s Thesis.

[4] Sharifi-Rad J., Sureda A., Tenore G.C., Daglia M., Sharifi-Rad M., Valussi M., Tundis R., Sharifi-Rad M., Loizzo M.R., Ademiluyi A.O. (2017). Biological activities of essential oils: From plant chemoecology to traditional healing systems. Molecules.

[5] Dijilani A., Dicko A., Bouayed J., Bohn T. (2012). The therapeutic benefits of essential oils. Nutrition, Well-Being and Health.

[6] De Colmenares N.G., Dellacassa E., Hasegawa M., Montes Guyot M.A., de Díaz A.M.P., Ringuelet J.A., Stashenko E.E., Tillet S., Virrueta A.V., Bandoni A.L. (2003). Los aceites esenciales. Los Recursos Vegetales Aromáticos en Latinoamérica. Su Aprovechamiento Industrial Para la Producción de Aromas y Sabores.

[7] Aramrueang N., Asavasanti S., Khanunthong A., Pan Z., Zhang R., Zicari S. (2019). Leafy Vegetables. Integrated Processing Technologies for Food and Agricultural By-Products.

[8] Manousi N., Sarakatsianos I., Samanidou V., Grumezescu A.M., Holban A.M. (2019). Extraction techniques of phenolic compounds and other bioactive compounds from medicinal and aromatic plants. Engineering Tools in the Beverage Industry. Volume 3: The Science of Beverages.

[9] Stratakos A.C., Koidis A., Preedy V. (2016). Methods for Extracting Essential Oils. Essential Oils in Food Preservation, Flavor and Safety.

[10] Aziz Z.A.A., Ahmad A., Setapar S.H.M., Karakucuk A., Azim M.M., Lokhat D., Rafatullah M., Ganash M., Kamal M.A., Ashraf G.M. (2018). Essential oils: Extraction techniques, pharmaceutical and therapeutic potential—A review. Curr. Drug Metab..

[11] Martínez A. (2003). Aceites Esenciales.

[12] Andrés M.F., González-Coloma A., Sanz J., Burillo J., Sainz P. (2012). Nematicidal activity of essential oils: A review. Phytochem. Rev..

[13] Barbosa P., Lima A.S., Vieira P., Dias L.S., Barroso J.G., Pedro L.G., Figueiredo A.C., Mota M. (2010). Nematicidal activity of essential oils and volatiles derived from Portuguese aromatic flora against the pinewood nematode, Bursaphelenchus xylophilus. J. Nematol..

[14] Wińska K., Mączka W., Łyczko J., Grabarczyk M., Czubaszek A., Szumny A. (2019). Essential oils as antimicrobial agents—myth or real alternative?. Molecules.

[15] Lang G., Buchbauer G. (2012). A review on recent research results (2008–2010) on essential oils as antimicrobials and antifungals. A review. Flavour Fragr. J..

[16] Yang Y., Isman M.B., Tak J.H. (2020). Insecticidal activity of 28 essential oils and a commercial product containing cinnamomum cassia bark essential oil against sitophilus zeamais Motschulsky. Insects.

[17] Nazzaro F., Fratianni F., Coppola R., De Feo V. (2017). Essential oils and antifungal activity. Pharmaceuticals.

[18] Batish D.R., Singh H.P., Kohli R.K., Kaur S. (2008). Eucalyptus essential oil as a natural pesticide. For. Ecol. Manag..

[19] Amorati R., Foti M.C., Valgimigli L. (2013). Antioxidant activity of essential oils. J. Agric. Food Chem..

[20] Blowman K., Magalhães M., Lemos M.F.L., Cabral C., Pires I.M. (2018). Anticancer Properties of Essential Oils and Other Natural Products. Evid.-Based Complement. Altern. Med..

[21] Adorjan B., Buchbauer G. (2010). Biological properties of essential oils: An updated review. Flavour Fragr. J..

[22] Pauli A., Schilcher H. (2004). Specific Selection of Essential Oil Compounds for Treatment of Children’s Infection Diseases. Pharmaceuticals.

[23] Gañán N.A. (2014). Extracción y fraccionamiento de biocidas de origen natural mediante el uso de fluidos supercríticos. Ph.D. Thesis.

[24] Moos G.P., Smith P.A.S., Tavernier D. (1995). Glossary of class names of organic compounds and reactivity intermediates based on structure. Pure Appl. Chem..

[25] Zhang L., Lu S. (2017). Overview of medicinally important diterpenoids derived from plastids. Mini Rev. Med. Chem..

[26] Koul O., Walia S., Dhaliwal G.S. (2008). Essential oils as green pesticides: Potential and constraints. Biopest. Int..

[27] Montoya-Cadavid G.D.J., Montoya-Cadavid G.D.J. (2010). Generalidades. Aceites Esenciales: Una Alternativa de Diversificación Para el Eje Cafetero.

[28] Riley M.B., Williamson M.R., Maloy O. (2002). Plant disease diagnosis. Plant Health Inst. Index.

[29] Buttimer C., McAuliffe O., Colin Hill R.P.R., O’Mahony J., Coffey A. (2017). Bacteriophages and Bacterial Plant Diseases. Front. Microbiol..

[30] Horst R.K. (2013). Bacteria. Westcott’s Plant Disease Handbook.

[31] Sobiczewski P. (2008). Bacterial diseases of plants: Epidemiology, diagnostics and control. Zemdirb.-Agric..

[32] Pandey A.K., Singh P., Palni U.T., Tripathi N.N. (2012). In-vitro antibacterial activities of the essential oils of aromatic plants against *Erwinia herbicola* (Lohnis) and *Pseudomonas putida* (Kris Hamilton). J. Serb. Chem. Soc..

[33] De Carvalho C.C.C.R., Caramujo M.J. (2008). Ancient Procedures for the High-Tech World: Health Benefits and Antimicrobial Compounds from the Mediterranean Empires. Open Biotechnol. J..

[34] Thacker J.R.M., Thacker J.R.M. (2002). A brief history of arthropod pest control. An Introduction to Arthropod Pest Control.

[35] Edris A.E. (2007). Pharmaceutical and Therapeutic Potentials of Essential Oils and Their Individual Volatile Constituents: A Review. Phytother. Res..

[36] Chapman A.C. (1903). LVII—Essential oil of hops. J. Chem. Soc. Trans..

[37] Hoffman C., Evans A.C. (1911). The uses of spices as preservatives. J. Ind. Eng. Chem..

[38] Aljaafari M., Alhosani M.S., Abushelaibi A., Lia K.S., Lim S.H.E., El-Shemy H. (2020). Essential oils: Partnering with antibiotics. Essential Oils—Oils of Nature.

[39] Yap P.S.X., Yiap B.C., Ping H.C., Lim S.H.E. (2014). Essential Oils, A New Horizon in Combating Bacterial Antibiotic Resistance. Open Microbiol. J..

[40] Burt S. (2004). Essential oils: Their antibacterial properties and potential applications in foods—A review. Int. J. Food Microbiol..

[41] Knobloch K., Pauli A., Iberl B., Weigand H., Weis N. (1989). Antibacterial and antifungal properties of essential oil components. J. Essent. Oil Res..

[42] Raveau R., Fontaine J., Sahraoui L.H. (2020). Essential Oils as Potential Alternative Biocontrol Products against Plant Pathogens and Weeds: A Review. Foods.

[43] Santiago M.B., Moraes T.D.S., Massuco J.E., Silva L.O., Lucarini R., da Silva D.F., Vieira T.M., Crotti A.E.M., Martins C.H.G. (2018). In vitro evaluation of essential oils for potential antibacterial effects against Xylella fastidiosa. J. Phytopathol..

[44] Arici S.E., Sanli A. (2014). Effect of some essential oils against Rhizoctonia solani and Streptomycetes scabies on potato plants in field conditions. Ann. Res. Rev. Biol..

[45] Iacobellis N.S., Lo Cantore P., Capasso F., Senatore F. (2005). Antibacterial activity of *Cuminum cyminum* L. and *Carum carvi* L. essential oils. J. Agric. Food Chem..

[46] Choi O., Cho S.K., Kim J. (2016). Biological evaluation of 32 different essential oils against Acidovorax citrulli, with a focus on *Cinnamomum verum* essential oil. Afr. J. Biotechnol..

[47] Tu Q.B., Wang P.Y., Sheng S., Xu Y., Wang J.Z., You S., Zhu A.H., Wang J., Wu F.A. (2020). Microencapsulation and Antimicrobial Activity of Plant Essential Oil Against Ralstonia solanacearum. Waste Biomass Valoriz..

[48] Paret M.L., Cabos R., Kratky B.A., Alvarez A.M. (2010). Effect of plant essential oils on Ralstonia solanacearum race 4 and bacterial wilt of edible ginger. Plant. Dis..

[49] Najar B., Pistelli L., Mancini S., Fratini F. (2020). Chemical composition and in vitro antibacterial activity of essential oils from different species of Juniperus (section Juniperus). Flavour Fragr. J..

[50] Mohamed A.A., Behiry S.I., Younes H.A., Ashmawy N.A., Salem M.Z.M., Márquez-Molina O., Barbabosa-Pilego A. (2019). Antibacterial activity of three essential oils and some monoterpenes against *Ralstonia solanacearum* phylotype II isolated from potato. Microb. Pathogen..

[51] Kokoskova B., Pouvova D., Pavela R. (2011). Effectiveness of plant essential oils against Erwinia amylovora, *Pseudomonas syringae* pv. syringae and associated saprophytic bacteria on/in host plants. J. Plant. Pathol..

[52] Githaiga B.M., Gathuru E.M., Waithaka P.N., Kiarie L.W. (2018). Determination of antibacterial activity of essential oils from mint (Mentha spicata) leaves on selected pathogenic bacteria. J. Drugs Pharmac. Sci..

[53] Daferera D.J., Ziogas B.N., Polissiou M.G. (2003). The effectiveness of plant essential oils on the growth of *Botrytis cinerea*, *Fusarium* sp. and *Clavibacter michiganensis* subsp. michiganensis. Crop. Prot..

[54] Mengulluoglu M., Soylu S. (2012). Antibacterial activities of essential oils extracted from medicinal plants against seed-borne bacterial disease agent. Acidovorax avenae subsp citrulli. Res. Crops.

[55] Mihajilov-Krstev T., Radnović D., Kitić D. (2010). Antimicrobial activity of Satureja L. essential oils against phytopathogenic bacteria Erwinia amylovora. Biol. Nyssana.

[56] Karami-Osboo R., Khodaverdi M., Ali-Akbari F. (2010). Antibacterial Effect of Effective Compounds of Satureja hortensis and Thymus vulgaris Essential Oils against Erwinia amylovora. J. Agric. Sci. Tech..

[57] Jafarpour M., Golparvar A.R., Lotfi A. (2013). Antibacterial activity of essential oils from Thymus vulgaris, Trachyspermum ammi and Mentha aquatica against Erwinia carotovora in vitro. J. Herb. Drug.

[58] Arraiza M.P., Gonzalez-Coloma A., Andres M.F., Berrocal-Lobo M., Dominguez-Nuñez J.A., Da Costa A.C., Navarro-Rocha J., Calderon-Guerrero C., El-Shemy H. (2018). Antifungal Effect of Essential Oils. Potential of Essential Oils.

[59] Isman M.B., Machial C.M., Rai M., Carpinella M. (2006). Pesticides based on plant essential oils: From traditional practice to commercialization. Advances in Phytomedicine—Naturally Occurring Bioactive Compounds, Volume 3.

[60] Tomazoni E.Z., Pauletti G.F., da Silva Ribeiro R.T., Moura S., Schwambach J. (2017). In vitro and in vivo activity of essential oils extracted from Eucalyptus staigeriana, Eucalyptus globulus and Cinnamomum camphora against Alternaria solani Sorauer causing early blight in tomato. Sci. Hort..

[61] Zheng J., Liu T., Guo Z., Zhang L., Mao L., Zhang Y., Jiang H. (2019). Fumigation and contact activities of 18 plant essential oils on Villosiclava virens, the pathogenic fungus of rice false smut. Sci. Rep..

[62] Kalagatur N.K., Nirmal Ghosh O.S., Sundararaj N., Mudili V. (2018). Antifungal activity of chitosan nanoparticles encapsulated with Cymbopogon martinii essential oil on plant pathogenic fungi Fusarium graminearum. Front. Pharmacol..

[63] Tzortzakis N.G., Economakis C.D. (2007). Antifungal activity of lemongrass (*Cympopogon citratus* L.) essential oil against key postharvest pathogens. Innov. Food Sci. Emerg. Technol..

[64] Gakuubi M.M., Maina A.W., Wagacha J.M. (2017). Antifungal Activity of Essential Oil of Eucalyptus camaldulensis Dehnh. against Selected *Fusarium* spp.. Int. J. Microbiol..

[65] Muchembled J., Deweer C., Sahmer K., Halama P. (2018). Antifungal activity of essential oils on two Venturia inaequalis strains with different sensitivities to tebuconazole. Environ. Sci. Pollut. Res..

[66] Santana O., Cabrera R., Gonzalez-Coloma A., Sanchez-Vioque R., De los Mozos-Pascual M., Rodriguez-Conde M.F., Laserna-Ruiz I., Usano-Alemany J., Herraiz D. (2012). Perfil químico y biológico de aceites esenciales de plantas aromáticas de interés agro-industrial en Castilla-La Mancha (España). Grasas Aceites.

[67] Tomazoni E.Z., Pansera M.R., Pauletti G.F., Moura S., Ribeiro R.T.S., Schwambach J. (2016). In vitro antifungal activity of four chemotypes of Lippia alba (Verbenaceae) essential oils against Alternaria solani (Pleosporeaceae) isolates. Ann. Braz. Acad. Sci..

[68] Terzi V., Morcia C., Faccioli P., Vale G., Tacconi G., Malnati M. (2007). In vitro antifungal activity of the tea tree (Melaleuca alternifolia) essential oil and its major components against plant pathogens. Lett. Appl. Microbiol..

[69] Duarte Y., Pino O., Infante D., Sánchez Y., Travieso M.D.C., Martínez B. (2013). Efecto in vitro de aceites esenciales sobre Alternaria solani Sorauer. Rev. Prot. Veg..

[70] Scalvenzi L., Yaguache-Camacho B., Cabrera-Martínez P., Guerrini A. (2016). Actividad antifúngica in vitro de aceites esenciales de Ocotea quixos (Lam.) Kosterm. y *Piper aduncum* L.. Bioagro.

[71] Zabka M., Pavela R., Slezakova L. (2009). Antifungal effect of Pimenta dioica essential oil against dangerous pathogenic and toxinogenic fungi. Ind. Crops Prod..

[72] Gandarilla-Pacheco F.L., Torres-Caraballo S., de Luna-Santillana E.J., Quintero-Zapata I., Arroyo-Gonzalez N. (2020). Efecto inhibitorio de aceites esenciales en el crecimiento micelial de *Penicillium digitatum* (pers.) sacc. aislado de naranja dulce (*Citrus sinensis* osbeck). Agrociencia.

[73] Villaverde J.J., Sevilla-Morán B., Sandín-España B., López-Goti C., Alonso-Prados J.L. (2014). Biopesticides in the framework of the European Pesticide Regulation (EC) No. 1107/2009. Pest Manag. Sci..

[74] Philogène B.J.R., Regnault-Roger C., Vincent C., Mundi-Prensa E., Regnault-Roger C., Philogène B.J.R., Vincent C. (2004). Productos fitosanitarios insecticidas de origen vegetal: Promesas de ayer y hoy. Biopesticidas de Origen Vegetal.

[75] Singh O., Rathore H.S., Nollet L.M.L., Nollet L.M.L., Rathore H.S. (2015). Biochemical pesticides. Biopesticides Handbook.

[76] Pavela R., Benelli G. (2016). Essential Oils as Ecofriendly Biopesticides? Challenges and Constraints. Trends Plant Sci..

[77] Chandler D. (2017). AMBER: Background on Biopesticides. Agriculture and Horticulture Development Board Research Project CP158.

[78] Isman M.B., Mundi-Prensa E., Regnault-Roger C., Philogène B.J.R., Vincent C. (2004). Problemas y perspectivas de comercialización de los insecticidas de origen vegetal. Biopesticidas de Origen Vegetal.

[79] Glare T.R., Nollet L.M.L., Rathore H.S. (2015). Types of biopesticides. Biopesticides Handbook.

